# SelfCoLearn: Self-Supervised Collaborative Learning for Accelerating Dynamic MR Imaging

**DOI:** 10.3390/bioengineering9110650

**Published:** 2022-11-04

**Authors:** Juan Zou, Cheng Li, Sen Jia, Ruoyou Wu, Tingrui Pei, Hairong Zheng, Shanshan Wang

**Affiliations:** 1 School of Physics and Optoelectronics, Xiangtan University, Xiangtan 411105, China; 2 Paul C. Lauterbur Research Center for Biomedical Imaging, Shenzhen Institutes of Advanced Technology, Chinese Academy of Sciences, Shenzhen 518055, China; 3 College of Information Science and Technology, Jinan University, Guangzhou 510631, China; 4 Guangdong Provincial Key Laboratory of Artificial Intelligence in Medicial Image Analysis and Application, Shenzhen 518055, China

**Keywords:** dynamic MR imaging, self-supervised learning, collaborative learning, reunderampling data augmentation, co-training loss

## Abstract

Lately, deep learning technology has been extensively investigated for accelerating dynamic magnetic resonance (MR) imaging, with encouraging progresses achieved. However, without fully sampled reference data for training, the current approaches may have limited abilities in recovering fine details or structures. To address this challenge, this paper proposes a self-supervised collaborative learning framework (SelfCoLearn) for accurate dynamic MR image reconstruction from undersampled k-space data directly. The proposed SelfCoLearn is equipped with three important components, namely, dual-network collaborative learning, reunderampling data augmentation and a special-designed co-training loss. The framework is flexible and can be integrated into various model-based iterative un-rolled networks. The proposed method has been evaluated on an in vivo dataset and was compared to four state-of-the-art methods. The results show that the proposed method possesses strong capabilities in capturing essential and inherent representations for direct reconstructions from the undersampled k-space data and thus enables high-quality and fast dynamic MR imaging.

## 1. Introduction

Deep learning-based dynamic magnetic resonance (MR) imaging has attracted substantial attention in recent years. It draws knowledge from big datasets via network training and then uses the trained network to reconstruct a dynamic image from the undersampled k-space data. Compared to the classical compressed sensing methods [1,2,3,4,5,6,7], deep learning-based methods have made encouraging performances and progresses.

Based on the reliance on the fully sampled dataset or not, existing methods for dynamic MR imaging can be roughly classified into two types [8,9,10]: fully-supervised methods and unsupervised methods. For the fully-supervised methods, data pairs are needed for the training of the neural networks between the corrupted/ undersampled data and the ground truth/fully sampled data [11,12,13,14,15,16,17,18]. In this category, different network structures and prior knowledge have been explored [19,20,21,22,23,24,25,26]. For example, Schlemper et al. proposed a cascade network architecture composed of an intermediate de-aliasing convolutional neural network (CNN) module and a data consistency layer [22]. Chen et al. applied bidirectional convolutional recurrent neural network (CRNN) with interleaved data consistency to accelerate MR imaging [23]. Chen et al. designed a parallel framework, including a time-frequency domain CRNN and an image domain CRNN to simultaneously exploit spatiotemporal correlations [24]. Wang et al. applied both k-space and spatial prior knowledge to accelerate MR imaging [25]. Ke et al. exploited the low rank priors (SLR-Net) [26]. The aforementioned methods have made great progress in accelerating dynamic MRI reconstruction. However, one major challenge of the above methods is that, in many practical imaging scenarios, obtaining high-quality fully sampled dynamic MR data is infeasible due to various factors, such as the physiological motions of patients and imaging speed restriction. Therefore, the requirement for fully sampled reference data of network training limits the wide application of supervised learning methods.

To address this problem, researchers have developed unsupervised learning methods to train models without fully sampled reference data [27,28,29,30]. For example, Jin et al. extended the framework of deep image prior [31] to dynamic non-Cartesian MRI [29]. Recently, Yaman et al. proposed a classical self-supervised learning strategy (SSDU) for static MR imaging [32], which divides the acquired undersampled data into two parts, of which one is treated as input data, and another is used as the supervisory signals [33]. Subsequently, Acar et al. applied SSDU to reconstruct dynamic MR images [30]. The above-mentioned works have made great contributions to unsupervised dynamic MR image reconstruction. Nevertheless, since the undersampled data have incomplete inherent representation compared to the fully sampled data, these works still have room to improve in recovering fine details or structures.

To boost the performances for accelerating dynamic MR imaging without fully sampled reference data, this paper proposes a self-supervised collaborative learning framework named the SelfCoLearn. The SelfCoLearn is based on the assumption that the latent representation of network predictions is consistent under different reundersampling data augmentation from the same data. The SelfCoLearn performs collaborative training of a dual-network using reundersampling data augmentation to explore more sufficient prior knowledge compared to a single network. Specifically, from undersampled k-space data, the reundersampling data augmentation operations are implemented to obtain two reundersampling inputs for a dual-network. In addition, dual networks are trained collaboratively with a special-designed co-training loss in an end-to-end manner. By using this collaborative training strategy, the proposed framework can possess strong capabilities in capturing essential and inherent representations from the undersamled k-space data in a self-supervised learning manner. Moreover, the proposed framework is flexible and can be integrated with various model-based iterative un-rolled networks [34] for dynamic MR imaging. In summary, the main contributions of this work can be expressed as follows:

1. We present a self-supervised collaborative learning framework with reundersampling data augmentation for accelerating dynamic MR imaging. The proposed framework is flexible and can be integrated with various model-based iterative un-rolled networks;
2. A co-training loss, including both undersampled consistency loss term and a contrastive consistency loss term, is designed to guide the end-to-end framework to capture essential and inherent representations from undersamled k-space data;
3. Extensive experiments are conducted to evaluate the effectiveness of the proposed SelfCoLearn with different model-based iterative un-rolled networks, with more promising results obtained compared to self-supervised methods.

The remainder of this paper is organized as follows: Section 2 states the dynamic MR imaging problem and the proposed SelfCoLearn with different backbone networks. Section 3 summarizes the comparison experiments and results to demonstrate the effectiveness of SelfCoLearn. Section 4 presents discussions about the impact of different backbone networks and loss functions. Section 5 concludes the work.

## 2. Methodology

### 2.1. Dynamic MR Imaging Formulation

The problem of dynamic MR imaging aims to estimate dynamic MR image sequences $x\in C^{N}$ from undersampled measurements $y\in C^{M}\left(M\ll N\right)$ in k-space. $N=N_{h}N_{W}T$ is a vector. $N_{h}$ and $N_{W}$ are the height and width of the frame, respectively. *T* represents the number of frames in each sequence. Thus, the imaging model is described as follows:

(1) y=Ax+e

where $e\in C^{M}$ is noise and A=PF is an undersampled Fourier encoding operator, F is 2D Fourier transform to each frame in the image sequence and P is the undersampled mask for each frame. In general, the reconstruction problem is formulated as the following unconstrained optimization problem:

(2) $$x^{*}=\arg\min_{x}\frac{1}{2}{∥Ax-y∥}_{2}^{2}+\lambda R\left(x\right)$$

where R(x) represents a prior regularization item on x, and λ is the weight of the regularization. $\frac{1}{2}{∥Ax-y∥}_{2}^{2}$ is the data fidelity item, which guarantees the reconstruction result to be consistent with the raw undersampled measurements.

For fully-supervised deep learning methods, it typically uses a CNN $f_{CNN}\left(y|\theta \right)$ as a regularization term R(x), by learning the mapping between corrupted/undersampled data and their corresponding fully sampled data with parameters θ. Its mathematical description can be given as:

(3) $$\theta^{*}=\arg\min_{\theta }\sum_{i=1}^{S}L\left(f_{CNN}\left(y_{i}|\theta \right),x_{i}^{ref}\right)$$

where *i* is the index of the subject in the training dataset, and *S* is its total number. $x_{i}^{ref}$ is the ground truth (fully sampled reference data) of the subject data *i*. L(·) denotes the loss function between the predicted output and the ground truth, which typically adopts the $l_{1}-$norm or $l_{2}-$norm.

### 2.2. The Overall Framework

**Figure 1:**
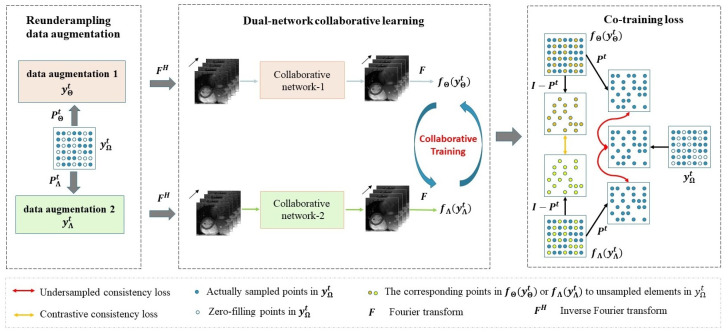
An overview of the proposed self-supervised collaborative training framework. A raw undersampled k-space data sequence $y_{\Omega }^{t}$ is undersampled from the fully sampled data using an undersampled mask $P^{t}$ retrospectively, and then two k-space data sequences $y_{\Theta }^{t}$ and $y_{\Lambda }^{t}$ are augmented from $y_{\Omega }^{t}$. In the considered scenario, $y_{\Theta }^{t}$ and $y_{\Lambda }^{t}$ are reundersampled from $y_{\Omega }^{t}$ using reundersampled mask $P_{\Theta }^{t}$ and $P_{\Lambda }^{t}$, respectively. Next, the two networks received inputs from zero-filling image sequences of $y_{\Theta }^{t}$ and $y_{\Lambda }^{t}$. The predicted image sequences of networks are transformed to the k-space data $f_{\Theta }\left(y_{\Theta }^{t}\right)$ and $f_{\Lambda }\left(y_{\Lambda }^{t}\right)$ by two-dimensional Fourier transform. Afterward, a co-training loss is calculated using $y_{\Omega }^{t}$, $f_{\Theta }\left(y_{\Theta }^{t}\right)$ and $f_{\Lambda }\left(y_{\Lambda }^{t}\right)$. The backbone reconstruction network can flexibly adopt different iterative un-rolled network, such as CRNN, k-t NEXT and SLR-Net. Collaborative network-1 and collaborative network-2 have the same network structure but different weight parameters $\theta_{\Theta }$ and $\theta_{\Lambda }$ respectively.

This work proposes a simple but effective self-supervised training framework for dynamic MR imaging, whose paradigm is shown in Figure 1. The proposed framework simultaneously trains two independent reconstruction networks, which have different inputs and different weight parameters. The backbone network can adopt various iterative un-rolled network, such as CRNN [23], k-t NEXT [21], and SLR-Net [26]. Based on the consistency between two networks’ prediction results, the network provides complementary information for the to-be-reconstructed dynamic MR images in its peer partner. The two networks will finally realize consistent reconstruction in the training process. Specifically, given a raw undersampled k-space data sequence $\Omega ={\left\{y_{\Omega }^{t}\right\}}_{t=1}^{T}$, the original k-space data $y_{\Omega }^{t}$ are reundersampled to construct a partial data points sequence ${\left\{y_{u}^{t}\right\}}_{t=1}^{T}$ as follows:

(4) $$y_{u}^{t}=P_{u}^{t}\left(y_{\Omega }^{t}\right),t=1,\ldots ,T,u=\Theta ,\Lambda$$

where *t* is the sequence index, *u* denotes the index of the two training sequences and $P_{u}^{t}$ is the undersampled mask for frame *t*. To achieve full use of all data points in $y_{\Omega }^{t}$ to learn representation, and ensure that each network can provide complementary information for the to-be-reconstructed dynamic MR images in its peer network, these training sequences are generated to adhere to the following data augmented principles: (1) The union of data points in two training sequences must be equal to the data $y_{\Omega }^{t}$, i.e., $y_{\Omega }^{t}=y_{\Theta }^{t}\cup y_{\Lambda }^{t}$. (2) The data points in two training sequences should be different, i.e., $y_{\Theta }^{t}\neq y_{\Lambda }^{t}$. (3) The training sequences should include most of the low frequency signals and part of the high frequency signals. Low frequency signals correspond to data points in the k-space center or close to it and high frequency signals to the outer parts of the k-space. Following these principles, the two training sequences contain different points in the high frequency region, and similar data points in the low frequency region. It should be noted that data reundersampling is necessary only during training, whereas the reconstructed images can be inferred from the test data directly.

### 2.3. Network Architectures

#### 2.3.1. Model-Driven Deep Learning with Image-Domain Regularization

In these settings, the common practice is to decouple Equation (2) into a regularization term and a data fidelity term via utilizing the variable splitting technique [22,23]. By introducing an auxiliary variable z=x, Equation (2) can be re-formulated as a penalty function [23], which can be expressed as follows:

(5) $$\arg\min_{x,z}\lambda R\left(z\right)+\frac{1}{2}{∥Ax-y∥}_{2}^{2}+\mu {∥x-z∥}_{2}^{2}$$

where μ denotes a penalty parameter. Equation (5) can then be solved iteratively via alternating minimization over z and x:

(6) $$z^{n}=\arg\min_{z}\lambda R\left(z\right)+\mu {∥x^{n-1}-z∥}_{2}^{2}$$

(7) $$x^{n}=\arg\min_{x}\frac{1}{2}{∥Ax-y∥}_{2}^{2}+\mu {∥x-z^{n}∥}_{2}^{2}$$

where $n\in \left\{1,2,\ldots ,N\right\}$ is the *n*th iteration, $x^{0}$ is the zero-filling image transformed from original undersampled measurement, $z^{n}$ denotes the intermediate reconstruction sequence, and $x^{n}$ denotes the final reconstruction sequence at each iteration. In Equation (7), the operation on the intermediate reconstruction sequence $z^{n}$ is a data consistency step [22]. The iterative optimization process in Equations (6) and (7) is unrolled into a neural network.

The CRNN [23] is a typical model-driven deep learning method with image-domain regularization for dynamic MR imaging [35]. A single iteration of the CRNN can be expressed as follows:

(8) $$x_{rnn}^{\left(n\right)}=x_{rec}^{\left(n-1\right)}+\mathrm{CRNN}\left(x_{rec}^{\left(n-1\right)}\right)$$

(9) $$x_{rec}^{\left(n\right)}=\mathrm{DC}\left(x_{rnn}^{\left(n\right)};y,\lambda \right)$$

where $x_{rnn}^{\left(n\right)}$ is the intermediate reconstruction sequence analogous to $z^{n}$ in Equation (6), and $x_{rec}^{\left(n\right)}$ denotes the final predicted result at each iteration analogous to $x^{n}$ in Equation (7). The regularization subproblem in Equation (6) is solved by using a convolutional recurrent neural network. The data consistency subproblem in Equation (7) is treated as a data consistency network layer, which uses the original sampled k-space data points to replace the corresponding data points in the reconstructed k-space data [22]. More details of CRNN layers can be found in Ref. [23].

#### 2.3.2. Model-Driven Deep Learning with Complementary Regularization

The complementary regularization is also an effective method for dynamic MR imaging. The k-t NEXT [21] is a typical model-driven deep learning method with complementary regularization [35], which exploits prior information in both combined spatial and temporal Fourier (x-f) domain and spatiotemporal image (x-t) domain. A single iteration of the k-t NEXT can be expressed as the following process:

(10) $$\rho^{\left(n\right)}=\mathrm{DC}\left(y_{base}\right)+\mathrm{xf}-\mathrm{CNN}\left(y_{rec}^{\left(n-1\right)}-y_{base}\right),$$

(11) $$x_{rec}^{\left(n\right)}=\mathrm{CRNN}\left(F_{f}^{H}\rho^{\left(n\right)};y_{0}\right),\;y_{rec}^{\left(n\right)}=F_{xy}x_{rec}^{\left(n\right)}$$

where $\rho^{\left(n\right)}$ denotes the intermediate reconstruction results in the x-f domain from xf-CNN at *n*th iteration, $x_{rec}^{\left(n\right)}$ denotes the reconstruction image sequence in the x-t domain at *n*th iteration, $y_{base}$ is the corresponding baseline signal, and $F_{xy}$ and $F_{f}^{H}$ denote, respectively, the Fourier transform in x-t domain and the inverse Fourier transform in x-f domain.

#### 2.3.3. Model-Driven Deep Learning with Low-Rank Regularization

Another widely-used prior regularization is low-rank based dynamic MR imaging, which applies low-rank priors as regularized terms. The SLR-Net [26] is a typical example of a model-driven deep learning method with low-rank regularization. In the SLR-Net, by introducing an auxiliary variable M, Equation (2) can be decoupled as the fidelity term, sparse regularization term, and the low rank regularization term:

(12) $$\arg\min_{x,M}\frac{1}{2}{∥Ax-y∥}_{2}^{2}+\lambda_{1}{∥Dx∥}_{1}+\lambda_{2}{∥M∥}_{*}$$

where *D* is a sparse transform in a certain sparse domain. M=Rx is a matrix (with size ($N_{h}\times N_{w}$, *T*)), in which each column corresponds to one frame in dynamic MR image sequence. *R* is a reshaping operator. ${∥M∥}_{*}$ is the nuclear norm. Previous works have proven that nuclear norm minimization is effective in low-rank matrix recovery [36]. More details of the iterative process in SLR-Net can be found in Ref. [26].

### 2.4. The Proposed Co-Training Loss

In this study, a co-training loss is defined to promote accurate dynamic MR image reconstruction in a self-supervised manner. The main idea of the co-training loss is to enforce the consistency not only between the reconstruction results and the original undersampled k-space data, but also between two network predictions. Compared with existing self-supervised methods with single network, the consistency between two network predictions is an additional regularization, which guides the dual-network to narrow the divergence and learn more correct information. Specifically, the co-training loss in SelfCoLearn, including an undersampled consistency loss term and a contrastive consistency loss term, is calculated to optimize the proposed framework.

Let $f_{SelfCoLearn}\left(y_{\Omega }^{t}\right)$ denote SelfCoLearn, $y_{\Omega }^{t}$ is the original undersampled k-space data. During training, two training sequences $y_{\Theta }^{t}$ and $y_{\Lambda }^{t}$ are generated from $y_{\Omega }^{t}$ following the data augmented principles in Section 2.2 as follows:

(13) $$y_{\Theta }^{t}=P_{\Theta }^{t}y_{\Omega }^{t},\;y_{\Lambda }^{t}=P_{\Lambda }^{t}y_{\Omega }^{t},$$

where $P_{\Theta }^{t}$ and $P_{\Lambda }^{t}$ are the reundersampled mask for $y_{\Omega }^{t}$. The undersampled consistency loss is mainly referred to the actually sampled k-space points in $y_{\Omega }^{t}$, which ensures that the corresponding sampled points in network prediction are consistent with the actually sampled k-space points in $y_{\Omega }^{t}$. The actually sampled points in these two network predictions are denoted as $y_{\Theta \to \Omega }^{t}$ and $y_{\Lambda \to \Omega }^{t}$, respectively. $y_{\Theta \to \Omega }^{t}$ and $y_{\Lambda \to \Omega }^{t}$ in these two network predictions can be written as:

(14) $$y_{\Theta \to \Omega }^{t}=P^{t}f\left(y_{\Theta }^{t}\right),\;y_{\Lambda \to \Omega }^{t}=P^{t}f\left(y_{\Lambda }^{t}\right),$$

where k-space data $f\left(y_{\Theta }^{t}\right)$ and $f\left(y_{\Lambda }^{t}\right)$ are transformed from the predicted image sequences of two networks, respectively. $P^{t}$ is the undersampled mask, which is applied to generate the raw undersampled k-space data $y_{\Omega }^{t}$ from the fully sampled data.

The Undersampled Consistency loss term is used to calculate the MSE between the actually sampled k-space points in $y_{\Omega }^{t}$ and those predicted by the network as follows:

(15) $$L_{UC}={∥y_{\Theta \to \Omega }^{t}-y_{\Omega }^{t}∥}_{2}^{2}+{∥y_{\Lambda \to \Omega }^{t}-y_{\Omega }^{t}∥}_{2}^{2}.$$

In the ideal case, when different reundersampled k-space data from the same data are set as inputs of the two networks, the networks’ predictions should approximate the fully-sampled reference data after network optimization. However, when fully sampled reference data are unavailable, these two networks can be trained only using the undersampled consistency loss, and they will be likely to generate different prediction results, which will lead to different reconstruction performances. As mentioned above, a contrastive consistency loss is defined to compute the MSE between two network predictions obtained by using different reundersampling inputs generated from the same data. Specially, the proposed contrastive consistency loss term mainly refers to the points in network predictions corresponding to unsampled k-space points in $y_{\Omega }^{t}$. Points ${\bar{y}}_{\Theta \to \Omega }^{t}$ and ${\bar{y}}_{\Lambda \to \Omega }^{t}$ in two network predictions $f\left(y_{\Theta }^{t}\right)$ and $f\left(y_{\Lambda }^{t}\right)$ can be expressed as follows:

(16) $${\bar{y}}_{\Theta \to \Omega }^{t}=\left(I-P^{t}\right)f\left(y_{\Theta }^{t}\right),\;{\bar{y}}_{\Lambda \to \Omega }^{t}=\left(I-P^{t}\right)f\left(y_{\Lambda }^{t}\right),$$

therefore, the Contrastive Consistency loss term is formulated as:

(17) $$L_{CC}={∥{\bar{y}}_{\Theta \to \Omega }^{t}-{\bar{y}}_{\Lambda \to \Omega }^{t}∥}_{2}^{2}.$$

combining the two loss terms, the final co-training loss function can be defined as follows:

(18) $$L_{co}=L_{UC}+\gamma L_{CC},$$

where γ is used to balance the weight parameter of the undersampled consistency loss and the contrastive consistency loss. During the testing phase, the undersampled data is used as input of the collaborative network-1 or collaborative network-2 to obtain the final reconstruction result.

## 3. Experimental Results

Extensive experiments have been performed to evaluate the effectiveness of SelfCoLearn. SelfCoLearn is compared with fully-supervised and self-supervised learning methods at different acceleration factors. Besides, SelfCoLearn with different backbone networks for dynamic MR imaging have been experimented. Then, the results of the ablation studies are reported to investigate the impacts of the undersampled consistency loss term and contrastive consistency loss term. Finally, reconstruction results with a different co-training loss calculated in different domains are reported to further evaluate the proposed SelfCoLearn.

### 3.1. Experimental Setup

#### 3.1.1. Dataset

The dataset includes fully sampled 2D+t complex-valued short-axis cardiac cine MR data collected on a 3T Siemens Magnetom Trio scanner from 101 healthy volunteers. T1-weighted FLASH sequence is utilized. Each scan includes single-slice FLASH acquisition from the volunteer with retrospectively electrocardiogram ECG-gating. Each volunteer needed to breath-hold for 15–20 s on each slice. The parameters of data acquisition include 24 receiving coils, FOV of 330 mm × 330 mm, acquisition matrix of 192 × 192, slice thickness of 6 mm, repetition time of 50 ms, and echo time of 3 ms. Each scan with a single slice covers the entire cardiac dynamic process with 25 temporal frames. This retrospective study was approved by local ethics committee and the informed consent was obtained from all of the involved volunteers. In the experiments, the set of scanned multi-coil MR data for each frame is transformed to a single-channel MRI by the adaptive reconstruction technique [37]. The corresponding k-space data to the single-channel MRI can be viewed as a fully sampled single-coil data. To enlarge the training dataset, we implement data augmentation strategies by shearing the single-channel complex-valued image along the dimensions of x, y, and t. After data augmentation, the dataset includes 6214 complex-valued data sequences of size 128 × 128 × 14. A total of 5950 cardiac MR data sequences were selected as the training dataset, 50 cardiac sequences were used as the validation dataset, and the remaining sequences were used for testing.

#### 3.1.2. Reundersampling K-Space Data Augmentation

In the proposed method, the fully sampled data are only used to generate the original undersampled k-space data $y_{\Omega }^{t}$ with a Cartesian retrospective undersampled mask $P^{t}$. Following the principles of training data augmentation in Section 2.2, $y_{\Omega }^{t}$ is augmented to two training sequences $y_{\Theta }^{t}$ and $y_{\Lambda }^{t}$ with two Cartesian reundersampled masks $P_{\Theta }^{t}$ and $P_{\Lambda }^{t}$. $P_{\Theta }^{t}$ with 2-fold acceleration is used for collaborative network-1, and $P_{\Lambda }^{t}$, which combines the complementary set of $P_{\Theta }^{t}$ with some low-frequency data points of $P^{t}$, is used for collaborative network-2.

#### 3.1.3. Evaluation Metrics

Reconstruction performances are evaluated by calculating mean-squared-error (MSE), peak-signal-to-noise ratio (PSNR), and structural similarity index (SSIM) [38] on magnitude images. The evaluation metrics are measured between the reconstruction image sequence Rec with the reference image sequence Ref as follows:

(19) $$\mathrm{MSE}={∥\mathrm{Ref}-\mathrm{Rec}∥}_{2}^{2}$$

(20) $$\mathrm{PSNR}=20\log_{10}\frac{MAX_{Ref}}{\sqrt{MSE}}$$

(21) $$\mathrm{SSIM}=\frac{\left(2\mu_{Ref}\mu_{Rec}+c_{1}\right)\left(2\sigma_{Ref,Rec}+c_{2}\right)}{\left(\mu_{Ref}^{2}+\mu_{Rec}^{2}+c_{1}\right)\left(\sigma_{Ref}^{2}+\sigma_{Rec}^{2}+c_{2}\right)}$$

where ${MAX}_{Ref}$ is the maximum possible value in the image. $\mu_{Ref}$ and $\mu_{Rec}$ are the averaged intensity values of the corresponding images. $\sigma_{Ref}$ and $\sigma_{Rec}$ are the variances. $c_{1}$ and $c_{2}$ are adjustable constants. $\sigma_{Ref,Rec}$ is the covariance. (details of SSIM index can be found in Ref. [38]).

#### 3.1.4. Model Configuration and Implementation Details

The proposed framework is flexible and can be integrated with various iterative un-rolled networks, such as CRNN, k-t NEXT and SLR-Net. Most of our experiments adopt CRNN as the backbone network. In detail, the network is composed of a bidirectional CRNN layer, three CRNN layers, a 2D CNN layer, a residual connection and a DC layer. For the bidirectional CRNN and CRNN layer, the convolution filter is set as 64 and the kernel size is set as 3. The 2D CNN layer has kernel size k=3 and convolution filter $N_{f}=2$. We use stride=1 and the padding is set to half of the filter size (rounded down). The DC layer is followed by the 2D CNN layer, which forces the actually sampled points in the predicted k-space data to be consistent with that in the input data.

For model training, the number of iteration steps is set to N=5. The batch size is set to 1. All training data and test data are normalized to the range of [0, 1]. The SelfCoLearn framework with CRNN and k-t NEXT is implemented in PyTorch 1.8.1, and that with SLR-Net is implemented in Tensorflow 2.2.0. The experiments are performed on an Nvidia Titan Xp GPU, with 12GB memory. SelfCoLearn is trained by Adam optimizer [39] with parameters $\beta_{1}=0.5$ and $\beta_{2}=0.999$. The learning rate is set to $10^{-4}$. The weight parameter γ in co-training loss is set to 0.01. It takes 52 h to train SelfCoLearn with CRNN and each cardiac MR data sequence takes roughly 0.5 s to get the reconstructed result.

### 3.2. Comparisons to State-of-the-Art Unsupervised Methods

To evaluate the proposed SelfCoLearn, we compared it with two self-supervised methods, SS-DCCNN and SS-CRNN, at different acceleration factors. It is worth noting that the state-of-the-art self-supervised method SSDU [32] was developed for static MR imaging. Ref. [30] adopted a similar self-supervised training manner as SSDU for dynamic MR imaging. They evaluated several backbone architectures for dynamic MR imaging including DCCNN and CRNN, whereas SSDU adopted ResNet as the backbone network. We choose two self-supervised learning methods SS-DCCNN and SS-CRNN [30] for comparison. In this experiment, the proposed SelfCoLearn selects the CRNN as the backbone network.

**Figure 2:**
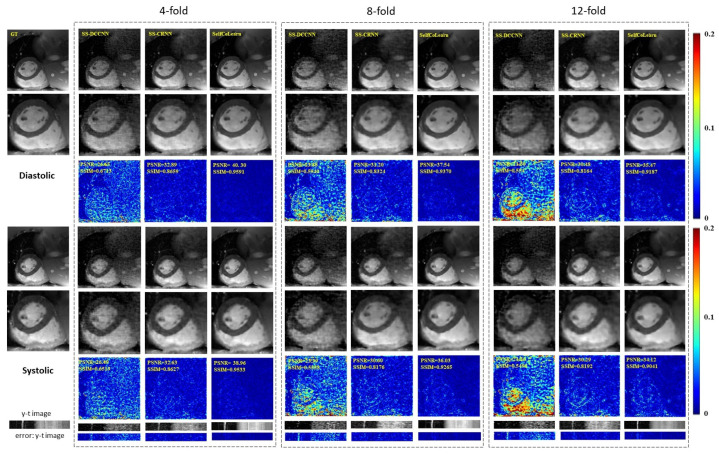
Reconstruction results of different self-supervised methods (SS-DCCNN, SS-CRNN, and SelfCoLearn) at 4-fold acceleration, 8-fold acceleration, and 12-fold acceleration. The first row and fourth row show the ground truth (fully sampled image) and the reconstruction images of the respective methods in the diastolic (the 10th frame of image sequence) and systolic (the 5th frame of image sequence), respectively. The second row and fifth row show their corresponding enlarged images in the heart regions. The third row and sixth row plot the error images of corresponding methods. The last two rows show y-t images (the 40th slice along the dimensions of y and t) and the corresponding error images.

Figure 2 plots the reconstruction results of different self-supervised methods at 4-fold acceleration, 8-fold acceleration, and 12-fold acceleration, respectively. The first row and fourth row show the ground truth (fully sampled image) and the reconstruction images of the respective methods in the diastolic and systolic at different accelerations, respectively (display range [0, 1]). The second row and fifth row show their corresponding enlarged images in the heart regions. The third row and sixth row plot the error images of the corresponding methods (display range [0, 0.2]). The y-t images at the 40th slice along the dimensions of y and t are shown in the seventh row. The corresponding error images of y-t images are plotted in the last row. From the visualization results, the proposed SelfCoLearn generates better reconstruction results than the two self-supervised methods, SS-DCCNN and SS-CRNN, at all acceleration factors. The reconstruction images of SelfCoLearn show finer structural details and more precise heart borders with fewer artifacts.

The quantitative results of these self-supervised methods are listed in Table 1. Similar conclusions can be obtained, showing that the SelfCoLearn achieves better quantitative performance than these self-supervised learning methods. Therefore, our collaborative learning strategy can effectively capture essential and inherent representations from undersampled k-space data directly.

**Figure 3:**
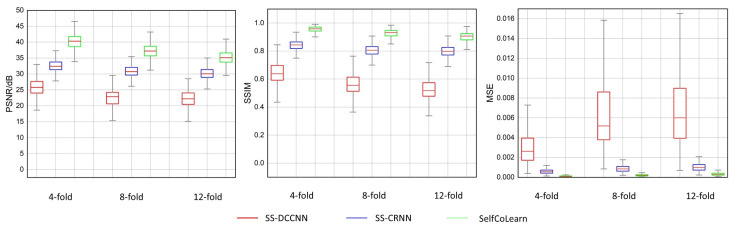
Box plots of different methods (SS-DCCNN, SS-CRNN, and SelfCoLearn) at 4-fold, 8-fold, and 12-fold accelerations are presented, which show the median and interquartile range of the PSNR, SSIM, and MSE on the cardiac cine test dataset.

Figure 3 shows the box plots displaying the median and interquartile range (25th–75th percentile) of the reconstruction results of different self-supervised methods on the test cardiac cine data at 4-fold acceleration, 8-fold acceleration, and 12-fold acceleration, respectively. The results in Figure 3 show that, for all dynamic cine sequences, the SelfCoLearn outperforms the two self-supervised learning methods (SS-DCCNN and SS-CRNN) at all three acceleration factors.

### 3.3. Comparisons to State-of-the-Art Supervised Methods

**Figure 4:**
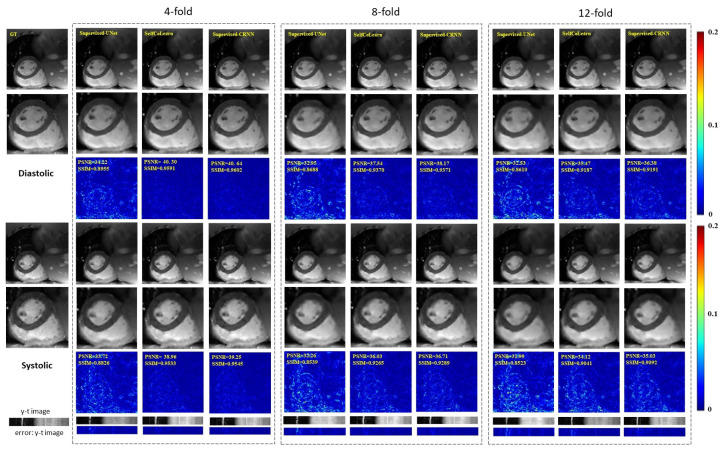
Reconstruction results of different methods (Supervised U-Net, SelfCoLearn, and Supervised CRNN) at 4-fold acceleration, 8-fold acceleration, and 12-fold acceleration. The first row and fourth row show the ground truth (fully sampled image) and the reconstruction images of respective methods in the diastolic (the 10th frame of the image sequence) and systolic (the 5th frame of the image sequence), respectively. The second row and fifth row show their corresponding enlarged images in the heart regions. The third row and sixth row plot the error images of the corresponding methods. The last two rows show y-t images (the 40th slice along the dimensions of y and t) and the corresponding error images.

We further compare our SelfCoLearn with different supervised methods, including supervised U-Net and supervised CRNN [23], at different acceleration factors. Figure 4 plots the reconstruction images of different methods at 4-fold acceleration, 8-fold acceleration, and 12-fold acceleration, respectively. The error images of SelfCoLearn indicate minor reconstruction errors than those of supervised U-Net.

In addition, the reconstruction results generated by SelfCoLearn are close to those of supervised CRNN at low acceleration factors. From the quantitative results in Table 2, the PSNR and SSIM of SelfCoLearn present 1.3% and 0.17% lower than those of supervised CRNN at 4-fold acceleration factors, respectively. At higher acceleration factors, such as 12-fold acceleration, the reconstructed images of SelfCoLearn become slightly blurred. Nevertheless, most of the structural details in the heart regions are still successfully restored by SelfCoLearn. The PSNR and SSIM of SelfCoLearn present 3.2% and 0.69% lower than those of supervised CRNN at 12-fold acceleration factors, respectively. Therefore, SelfCoLearn can achieve comparable reconstruction performance with baseline fully-supervised methods via self-supervised dual-network collaborative learning.

## 4. Discussion

### 4.1. Network Backbone Architectures

**Figure 5:**
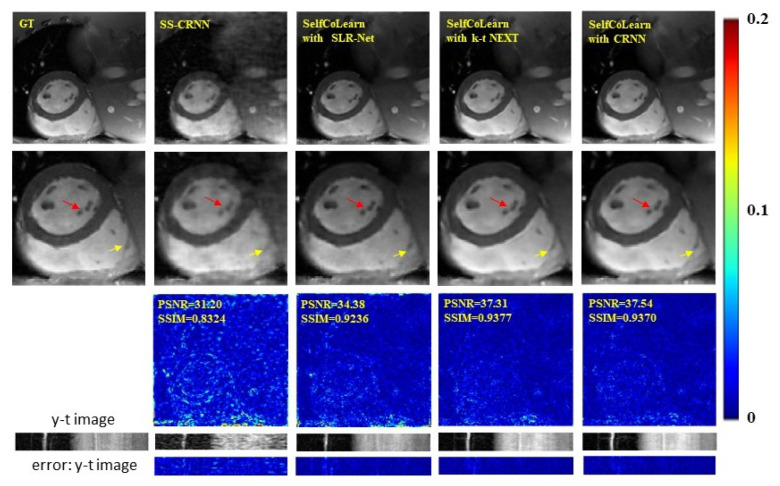
Reconstruction results of SS-CRNN and the proposed SelfCoLearn with SLR-Net, k-t NEXT, and CRNN backbone networks at 8-fold acceleration. The first row shows ground truth (fully sampled image), the reconstruction images of SS-CRNN and the proposed self-supervised learning strategy with SLR-Net, k-t NEXT, and CRNN (10th frame). The second row shows their enlarged images in the heart regions. The third row plots the error images of these two methods. The last two rows show the y-t images (the 40th slice along the dimensions of y and t) and the corresponding error images.

In this section, we explore the reconstruction results of the proposed self-supervised learning strategy with different backbone networks for dynamic MR imaging. The experiments are conducted using SLR-Net [26], k-t NEXT [21], and CRNN [23] at 8-fold acceleration. The reconstruction results with different backbone networks are exhibited in Figure 5 and Table 3. Compared with SS-CRNN [11], the proposed SelfCoLearn can achieve better results regardless of the utilized backbone network. Among the three different backbone networks, SLR-Net generates worse results than k-t NEXT and CRNN. The reason for this phenomenon may be that SLR-Net needs to learn a singular value threshold, and the absence of the fully sampled reference data causes the learned singular value threshold to be suboptimal. However, the proposed self-supervised learning strategy with SLR-Net still obtains acceptable reconstruction results. The qualitative results in Figure 5 clearly show that SelfCoLearn can better restore the structural details and achieve clearer reconstructed MR images (especially in the heart regions around the red and yellow arrows) than SS-CRNN. The quantitative results also indicate more accurate reconstructions achieved by the proposed SelfCoLearn. These results indicate that our proposed self-supervised learning framework is flexible, and it can achieve promising reconstruction results with various iterative un-rolled networks for dynamic MR imaging.

### 4.2. Co-Training Loss Function

**Figure 6:**
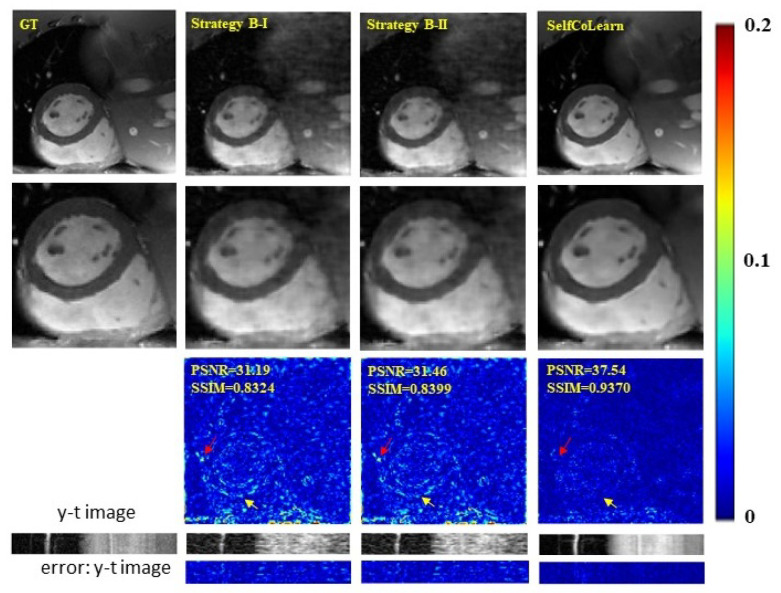
Ablation studies utilizing different training strategies at 8-fold acceleration. The first row shows the ground truth (fully sampled image), and the reconstruction images of strategy B-I, strategy B-II, and proposed SelfCoLearn (10th frame). The second row shows their enlarged images in the heart regions. The third row plots the error images of respective methods. The last two rows show y-t images (the 40th slice along the dimensions of y and t) and the corresponding error images.

In this section, we investigate the utility of the designed co-training loss function. The backbone network in these experiments adopts CRNN. Different training strategies at 8-fold acceleration are utilized. Strategy B-I: a single reconstruction network is trained in self-supervised manner. Only the loss function between the output $f\left(y_{\Theta }^{t}\right)$ of network and $y_{\Lambda }^{t}$ is used to train the network. Strategy B-II: a strategy similar to B-I but the loss function here is calculated between the output $f\left(y_{\Theta }^{t}\right)$ of the network and the original undersampled k-space data $y_{\Omega }^{t}$. SelfCoLearn: two networks are trained collaboratively with $L_{UC}$ and $L_{CC}$, and the two collaborative networks adopt the same backbone network as that in strategy B-I. Reconstruction images of methods utilizing the different training strategies are plotted in Figure 6. Quantitative results are listed in Table 4. From both qualitative and quantitative results, we can observe that SelfCoLearn (training two networks collaboratively with both loss terms) achieves the best performance (especially in the heart regions around the red and yellow arrows). In particular, the contrastive consistency loss term results in a large reconstruction performance improvement. For example, PSNR is improved from 31.04 dB (Strategy B-II) to 37.27 dB (SelfCoLearn).

### 4.3. Loss Functions

**Figure 7:**
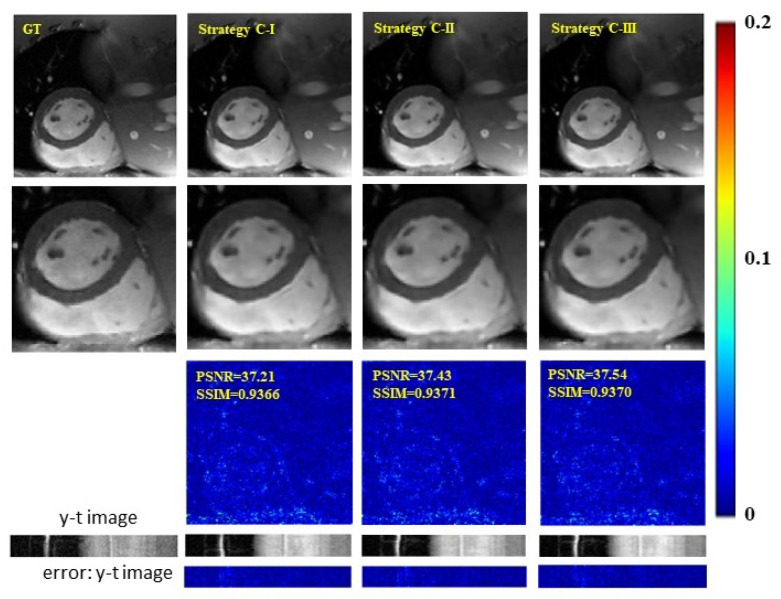
Effects of loss functions calculated in different domains on the reconstruction results at 8-fold acceleration. The first row shows ground truth (fully sampled image), the reconstruction results of models utilizing Strategy C-I, C-II and C-III (10th frame). The second row shows their enlarged images in the heart regions. The third row plots their error images of respective strategies. The last two rows show y-t images (the 40th slice along the dimensions of y and t) and the corresponding error images.

In this section, we inspect the effects of loss functions. The backbone network in these experiments adopts CRNN. Reconstruction results at 8-fold acceleration are given in Figure 7 and Table 5. Three strategies utilizing different loss function settings are investigated. In Strategy C-I, two networks are trained collaboratively with $L_{UC}$ and $L_{CC}$, in which $L_{UC}$ is calculated in the x-t domain, and $L_{CC}$ is calculated in the k-space domain. In Strategy C-II, both $L_{UC}$ and $L_{CC}$ are calculated in the x-t domain. In Strategy C-III, both $L_{UC}$ and $L_{CC}$ are calculated in the k-space domain. From both qualitative and quantitative results, we can observe that the influence of utilizing different loss function settings on the reconstruction performance is insignificant. All the other experiments in this work adopt the setting of strategy C-III.

## 5. Conclusions

In our work, we propose a self-supervised collaborative training framework to boost the image reconstruction performance for accelerating dynamic MR imaging. Specifically, two independent reconstruction networks are trained collaboratively with different inputs, which are augmented from the same k-space data. To guide the dual-network in capturing the detailed structural features and spatiotemporal correlations in dynamic image sequences, a co-training loss function is designed to promote the consistency between network predictions to provide complementary information for the to-be-reconstructed dynamic MR images. The proposed framework is flexible and can be integrated with various iterative un-rolled networks. In addition, the proposed method has been comprehensively evaluated on a cardiac cine dataset. The quantitative and qualitative results indicate that SelfCoLearn possesses strong capabilities in capturing essential and inherent representations directly from the undersampled k-space data and thus enable high-quality and fast dynamic MR imaging.

## Figures and Tables

**Table 1:** Quantitative reconstruction results of different self-supervised methods (SS-DCCNN, SS-CRNN, and SelfCoLearn) at 4-fold, 8-fold, and 12-fold acceleration factors (mean ± std).

AF	Methods	Training Pattern	PSNR (dB)	SSIM	MSE ($\times 10^{-4}$)
	SS-DCCNN	Self-supervised	25.81 ± 2.86	0.6409 ± 0.0739	32.81 ± 24.85
4-fold	SS-CRNN	Self-supervised	32.49 ± 1.79	0.8383 ± 0.0387	6.14 ± 2.62
	SelfCoLearn	Self-supervised	40.34 ± 2.69	0.9536 ± 0.0239	1.11 ± 0.72
	SS-DCCNN	Self-supervised	22.56 ± 2.71	0.5615 ± 0.0732	67.87 ± 49.27
8-fold	SS-CRNN	Self-supervised	30.81 ± 1.77	0.8015 ± 0.0427	9.02 ± 3.75
	SelfCoLearn	Self-supervised	37.27 ± 2.40	0.9243 ± 0.0338	2.17 ± 1.22
	SS-DCCNN	Self-supervised	22.17 ± 2.76	0.5270 ± 0.0702	74.89 ± 54.96
12-fold	SS-CRNN	Self-supervised	30.14 ± 1.78	0.7943 ± 0.0444	10.54 ± 4.40
	SelfCoLearn	Self-supervised	35.19 ± 2.24	0.8985 ± 0.0399	3.44 ± 1.78

**Table 2:** Quantitative reconstruction results of different methods (Supervised U-Net, Supervised CRNN and SelfCoLearn) at 4-fold, 8-fold, and 12-fold acceleration factors (mean ± std).

AF	Methods	Training Pattern	PSNR (dB)	SSIM	MSE ($\times 10^{-4}$)
	U-Net	Supervised	33.77 ± 1.96	0.8698 ± 0.0391	4.66 ± 2.22
4-fold	SelfCoLearn	Self-supervised	40.34 ± 2.69	0.9536 ± 0.0239	1.11 ± 0.72
	CRNN	Supervised	40.89 ± 2.90	0.9553 ± 0.0237	1.01 ± 0.68
	U-Net	Supervised	32.63 ± 1.97	0.8329 ± 0.0456	6.06 ± 2.88
8-fold	SelfCoLearn	Self-supervised	37.27 ± 2.40	0.9243 ± 0.0338	2.17 ± 1.22
	CRNN	Supervised	38.09 ± 2.52	0.9269 ± 0.0342	1.83 ± 1.07
	U-Net	Supervised	31.96 ± 1.88	0.8315 ± 0.0478	6.99 ± 3.03
12-fold	SelfCoLearn	Self-supervised	35.19 ± 2.24	0.8985 ± 0.0399	3.44 ± 1.78
	CRNN	Supervised	36.32 ± 2.29	0.9048 ± 0.0392	2.67 ± 1.42

**Table 3:** Quantitative results of SS-CRNN and SelfCoLearn with different backbone networks at 8-fold acceleration (mean ± std).

Methods	Training Pattern	PSNR (dB)	SSIM	MSE ($\times 10^{-4}$)
SS-CRNN	Self-supervised	30.81 ± 1.77	0.8015 ± 0.0427	9.02 ± 3.75
SelfCoLearn with SLR-Net	Self-supervised	33.58 ± 2.24	0.9001 ± 0.0369	5.57 ± 10.48
SelfCoLearn with k-t Next	Self-supervised	36.95 ± 2.39	0.9226 ± 0.0343	2.34 ± 1.32
SelfCoLearn with CRNN	Self-supervised	37.27 ± 2.40	0.9243 ± 0.0338	2.17 ± 1.22

**Table 4:** Quantitative results of reconstruction models utilizing different training strategies at 8-fold acceleration (mean ± std).

Methods	Single-Net	Parallel-Net	$L_{UC}$	$L_{CC}$	PSNR (dB)	SSIM	MSE ($\times 10^{-4}$)
Strategy B-I	√	×	×	×	30.81 ± 1.77	0.8015 ± 0.0427	9.02 ± 3.75
Strategy B-II	√	×	√	×	31.04 ± 1.74	0.8102 ± 0.0411	8.53 ± 3.50
SelfCoLearn	×	√	√	√	37.27 ± 2.40	0.9243 ± 0.0338	2.17 ± 1.22

**Table 5:** Quantitative results of methods utilizing different loss function strategies at 8-fold acceleration (mean ± std).

Methods	$L_{UC}$	$L_{CC}$	PSNR (dB)	SSIM	MSE ($\times 10^{-4}$)
Strategy C-I	x-t domain	k-space	37.00 ± 2.35	0.9230 ± 0.0344	2.30 ± 1.29
Strategy C-II	x-t domain	x-t domain	37.20 ± 2.37	0.9235 ± 0.0343	2.20 ± 1.22
Strategy C-III	k-space	k-space	37.27 ± 2.40	0.9243 ± 0.0338	2.17 ± 1.22

## Data Availability

The source code will be available publicly upon publication of the manuscript.
